# Treating sarcoidosis-associated progressive multifocal leukoencephalopathy with infliximab

**DOI:** 10.1093/braincomms/fcab292

**Published:** 2021-12-16

**Authors:** Sina C. Rosenkranz, Vivien Häußler, Manuela Kolster, Anne Willing, Jakob Matschke, Christoph Röcken, Klarissa Stürner, Frank Leypoldt, Eva Tolosa, Manuel A. Friese

**Affiliations:** Institute of Neuroimmunology and Multiple Sclerosis, University Medical Center Hamburg-Eppendorf, Germany; Department of Neurology, University Medical Center Hamburg-Eppendorf, Germany; Institute of Neuroimmunology and Multiple Sclerosis, University Medical Center Hamburg-Eppendorf, Germany; Department of Neurology, University Medical Center Hamburg-Eppendorf, Germany; Department of Immunology, University Medical Center Hamburg-Eppendorf, Germany; Institute of Neuroimmunology and Multiple Sclerosis, University Medical Center Hamburg-Eppendorf, Germany; Institute of Neuropathology, University Medical Center Hamburg-Eppendorf, Germany; Institute of Pathology, University Medical Center Schleswig-Holstein, Kiel, Germany; Neuroimmunology, Institute of Clinical Chemistry, University Medical Center Schleswig-Holstein, Kiel, Germany; Department of Neurology, University Medical Center Schleswig-Holstein and Kiel University, Kiel, Germany; Neuroimmunology, Institute of Clinical Chemistry, University Medical Center Schleswig-Holstein, Kiel, Germany; Department of Neurology, University Medical Center Schleswig-Holstein and Kiel University, Kiel, Germany; Department of Immunology, University Medical Center Hamburg-Eppendorf, Germany; Institute of Neuroimmunology and Multiple Sclerosis, University Medical Center Hamburg-Eppendorf, Germany

**Keywords:** progressive multifocal leukoencephalopathy (PML), sarcoidosis, infliximab, regulatory T cells (Tregs), CD4^+^ T cells

## Abstract

Although most of the progressive multifocal leukoencephalopathy cases in sarcoidosis patients are explained by the treatment with immunosuppressive drugs, it is also reported in treatment-naive sarcoidosis patients, which implies a general predisposition of sarcoidosis patients for progressive multifocal leukoencephalopathy. Indeed, it was shown that active sarcoidosis patients have increased regulatory T cell frequencies which could lead to a subsequent systemic immunosuppression. However, if sarcoidosis with systemic changes of T cell subsets frequencies constitute a risk factor for the development of progressive multifocal leukoencephalopathy, which could then be counteracted by sarcoidosis treatment, is not known. In this cohort study, we included, characterized and followed-up six patients with bioptically confirmed definite progressive multifocal leukoencephalopathy and definite or probable sarcoidosis presenting between April 2013 and January 2019, four of them had no immunosuppressive therapy at the time of developing first progressive multifocal leukoencephalopathy symptoms. Analysis of immune cell subsets in these patients revealed significant imbalances of CD4^+^ T cell and regulatory T cell frequencies. Due to the progression of progressive multifocal leukoencephalopathy in four patients, we decided to treat sarcoidosis anticipating normalization of immune cell subset frequencies and thereby improving progressive multifocal leukoencephalopathy. Notably, by treatment with infliximab, an antibody directed against tumour necrosis factor-α, three patients continuously improved clinically, JC virus was no longer detectable in the cerebrospinal fluid and regulatory T cell frequencies decreased. One patient was initially misdiagnosed as neurosarcoidosis and died 9 weeks after treatment initiation due to aspiration pneumonia. Our study provides insight that sarcoidosis can lead to changes in T cell subset frequencies, which predisposes to progressive multifocal leukoencephalopathy. Although immunosuppressive drugs should be avoided in progressive multifocal leukoencephalopathy, paradoxically in patients with sarcoidosis treatment with the immunosuppressive infliximab might restore normal T cell distribution and thereby halt progressive multifocal leukoencephalopathy progression.

## Teaser

Rosenkranz *et al.* report that active systemic sarcoidosis can result in an increase of regulatory T-cell frequencies and concomitant decrease of CD4+ T-cell numbers that predisposes to progressive multifocal leukoencephalopathy. Under infliximab treatment of sarcoidosis, regulatory T-cell frequencies decreased which was associated with continuous clinical improvement.

## Graphical Abstract

**Graphical Abstract:**
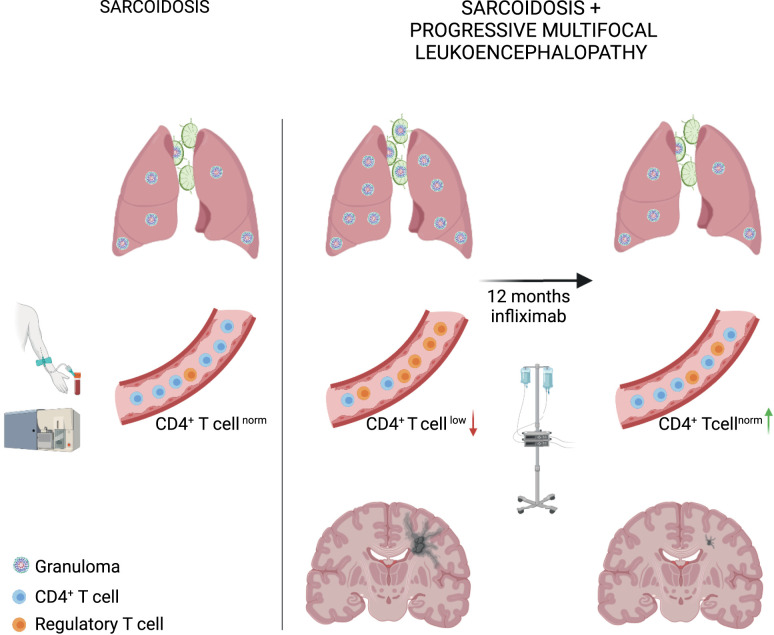

## Introduction

Progressive multifocal leukoencephalopathy (PML) is a devastating demyelinating disease caused by reactivation of JC virus (JCV)^1^ due to severe primary or acquired immunodeficiency or by treatment with immunosuppressive drugs, but it is also reported in patients with minimal or occult immunodeficiency.^2,3^

Sarcoidosis is a systemic granulomatous disease, which is characterized by non-caseating epithelioid granulomas mainly affecting the lung and lymph nodes.^4^ Associations of PML and sarcoidosis are described^5,6^; however, most patients were taking immunosuppressive drugs like steroids and steroid-sparing agents. Yet, PML has also been observed to develop in treatment-naive sarcoidosis patients^5,7,8^ and even led to the discovery of underlying occult sarcoidosis.^9–11^

Active sarcoidosis is characterized by an accelerated inflammation accumulating in granulomas that can result in increased regulatory T cell (Treg) frequencies and subsequent systemic immunosuppression,^12^ which might predispose to an increased risk of opportunistic infections. We hypothesized that sarcoidosis itself causes alterations in T cell subset frequencies predisposing to PML and that immune homeostasis might be restored by sarcoidosis-targeted therapy.

Here, we report on six patients with brain bioptically confirmed definite PML^13^ and systemic sarcoidosis. Comparison of the immune cell compartments to sarcoidosis patients without PML revealed an increase in Treg frequencies and a concomitant decrease of CD4^+^ T cell numbers. Four patients with progressive PML were experimentally treated with infliximab, which is an established therapy for sarcoidosis. Under this treatment, we could observe a continuous clinical and radiological improvement as well as reduced Treg frequencies and clearance of JCV DNA in the CSF in three patients.

## Materials and methods

### Patient recruitment

All patients that were diagnosed with PML between April 2013 and January 2019 at the University Medical Center Hamburg-Eppendorf in Hamburg, Germany or the University Medical Center Schleswig-Holstein in Kiel, Germany without any explanation for immunosuppression were screened for definite (bioptically confirmed) or probable sarcoidosis and followed prospectively (prospective cohort; *n* = 5). Additionally, we retrospectively screened all patients with PML between 2010 and 2013 at participating centres and identified one patient with probable sarcoidosis (*n* = 1, retrospective cohort). Follow-up was performed within the clinical routine. As controls, we recruited four patients with definite sarcoidosis without clinical or radiological signs of PML (*n* = 4) at the University Medical Center Hamburg-Eppendorf (*n* = 3) or the Medical Clinic and Research Center Borstel in Borstel, Germany (*n* = 1) within the clinical routine. Healthy controls for comparing Treg frequencies were recruited at the University Medical Center Hamburg-Eppendorf (*n* = 10; seven males and three females).

### Patient consents

All participants provided written informed consent and the study was approved by the local ethics committee (Board of Physicians, Hamburg, Nos PV4405 and PV3392).

### Human blood samples

Concomitant immunosuppression at the time of sampling was recorded. Leukocyte counts were determined in whole blood samples from all patients within clinical routine work-up. Immune cell subpopulations were determined by flow cytometric analysis of fresh blood; except in one case where frozen peripheral blood mononuclear cells (Patient #1) were used. Here, numbers of total lymphocytes and B cells were also determined automatically. The absolute counts of the subpopulations were calculated according to the number of leukocytes per microlitre of blood. Soluble interleukin-2 (IL2) receptor (sIL2R) was determined in the serum using standard commercial test kits (Roche Cobas). One sample was measured in nanogram per millilitre and converted to units per millilitre for comparison. JCV DNA was determined at the Institute of Virology, University Medical Center Heinrich-Heine Düsseldorf, Germany (detection limit of 100 copies ml^−1^) and the National Institute of Neurological Disorders and Stroke, Bethesda, MD, USA (detection limit of 10 copies ml^−1^).

### Flow cytometry

One hundred microlitres of peripheral EDTA blood were stained with fluorochrome-labelled antibodies directed against CD45, CD19, CD3, CD4, CD8, CD56, CD25 and CD127 (all from Biolegend or BD Biosciences) for 30 min at room temperature. Samples were subsequently lysed with BD FACS Lysing Solution, fixed in 1% PFA. Treg cells were identified by the high expression of CD25 and absence of CD127 within the CD4^+^ T cell compartment. For intracytoplasmic cytokine staining, 100 µl heparin blood was stimulated with 250 ng ml^−1^ PMA and 5 µg ml^−1^ ionomycin to broadly assess immune function. Cells were cultured for 5 h in a serum-free medium (X-Vivo15) in the presence of 10 µg ml^−1^ Brefeldin A. After stimulation, cells were incubated with antibodies directed against the surface markers CD3, CD4 and CD8, permeabilized and stained with antibodies directed against interferon-γ (IFN-γ) and tumour necrosis factor-α (TNF-α). Samples were measured on an LSR Fortessa flow cytometer (BD Bioscience) and data were analysed using FlowJo 10.0.8 software (TreeStar).

### Statistical analysis

Prism 8 software (GraphPad Software) for Mac was used for data analysis; data are presented as mean ± SEM. Data were analysed for normal distribution using the Shapiro–Wilk test. Differences between sarcoidosis patients with PML (SAR-PML) and without PML (SAR-CON) were determined using an unpaired, two-tailed Student's *t*-test. For comparisons before and after treatment with infliximab in SAR-PML patients, paired two-tailed Student’s *t*-test was used. For all statistical tests, *P*-value, *t*-value and degrees of freedom are reported. Significant results are indicated by *P < 0.05, **P < 0.01, ***P < 0.001, ****P < 0.0001.

### Data availability statement

All data generated or analysed during this study are included in this published article. Further data collected between the specified dates of follow-up are available from the corresponding author on reasonable request. Individual de-identified participant data (demographical data, clinical data, laboratory parameters) are shared. Data will be available for 10 years and can be accessed by S.C.R., V.H., F.L. and M.A.F.

## Results

### Diagnosis of PML in a patient with sarcoidosis

**Figure 1:**
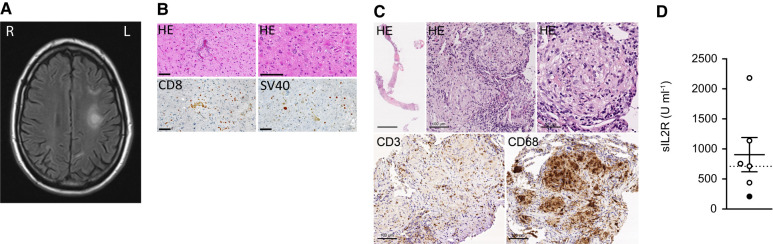
**Characterization of PML patients.** (**A**) MRI of Patient #1 at presentation with Balint’s syndrome showing hyperintense signals parietooccipital right and in the inner capsule left in T_2_-weighted scans. (**B**) Neuropathological analyses of brain biopsy (Patient #1) by immunostaining with haematoxylin/eosin (HE) showed myelin pallor, reactive gliosis and atypical astrocytes with pleomorphic nuclei. Staining with antibodies directed against CD8 revealed sparse perivascular CD8-positive lymphocytic infiltration. Anti-SV40 staining identified the infected oligodendrocytes and confirmed PML diagnosis. Scale bar: 100 µm. (**C**) A punch biopsy obtained from the lung of Patient #5 showed non-necrotizing epithelioid granulomas in HE staining, which enclosed only scatted CD3-positive T cells and CD68-positive epithelioid cells. Scale bar: 50 and 100 µm. (**D**) Serum sIL2 receptor (sIL2R) is elevated in sarcoidosis patients with PML (SAR-PML). SAR-PML (*n* = 6) patients are plotted for their serum sIL2R levels with grid line showing the upper limit of the reference range at <710 U ml^−1^. Data from Patient #6 who received corticosteroids at blood sampling is presented as a filled circle. Data are presented as mean value ± SEM.

In January 2013, a 49-year-old male (Patient #1) was admitted to the University Medical Center Hamburg-Eppendorf due to progressive visual problems. He reported a prior episode of similar symptoms with spontaneous recovery 10 months earlier. He had a medical history of systemic sarcoidosis (Stage II = lymphadenopathy and parenchymal disease), histologically proven by a mediastinal lymph node biopsy in 2001, which led to a treatment with corticosteroids for 18 months (2001–02). However, he was not taking any immunosuppressive medication at the time of developing neurological symptoms in 2012 and 2013. Neurological examination revealed a Balint’s syndrome, which consists of simultanagnosia, optic ataxia and oculomotor apraxia. Cranial MRI showed increased signal intensities in T_2_-weighted scans in the parietooccipital area and in the inner capsule and no gadolinium enhancement in T_1_-weighted scans (Fig. 1A). CSF analysis revealed an elevated protein level of 721 mg l^−1^ without pathological cell counts or isolated oligoclonal bands. PCR analysis for JCV DNA in the CSF was negative (detection limit 100 copies ml^−1^). Due to the rapid progression of neurological symptoms with enlarging lesions and hemiparesis, a brain biopsy was performed in April 2013 leading to the diagnosis of PML (Fig. 1B). Repeated analysis of the CSF for JCV DNA by PCR confirmed the diagnosis with 20 copies ml^−1^ (detection limit 10 copies ml^−1^).

In order to assess whether PML could be explained by a dysregulated immune system caused by systemic sarcoidosis, we analysed the immune cell composition in the peripheral blood by flow cytometry, which revealed decreased numbers of CD4^+^ T cells (139.9 µl^−1^; reference: 500–1350 µl^−1^), and an increased frequency of Tregs (13% of CD4^+^ T cells; mean of 10 healthy controls 6.1%, SEM ± 1.007). Evaluation of the inflammatory activity of the sarcoidosis revealed increased serum sIL2R levels at 1840 U ml^−1^ (reference: <710 U ml^−1^) and increased tracer uptake of mediastinal and retroperitoneal lymph nodes in fluorodeoxyglucose-PET (FDG-PET) scan, leading to the diagnosis of active systemic sarcoidosis. As the patient was not taking any immunosuppressive medication and we excluded other known systemic immunodeficiencies or malignancies, we hypothesized that active systemic sarcoidosis had resulted in changes of immune cell frequencies that compromised JCV control and consecutively led to the development of PML.

### Patient cohort and clinical presentation

To confirm this single patient observation, both participating large University Hospitals (Hamburg and Kiel, Germany) started to routinely screen all patients with a diagnosis of PML for concomitant sarcoidosis. Between April 2013 and January 2019, we identified and prospectively followed five patients (including Patient #1) with bioptically confirmed definite PML^13^ and underlying systemic sarcoidosis. Characterization of PML patients with sarcoidosis is shown in Table 1.

**Table 1:** PML patient cohort characteristics and clinical presentation

Sex	Patient 1	Patient 2	Patient 3^a^	Patient 4	Patient 5	Patient 6
	Male	Male	Male	Male	Male	Male
Age (years)^b^	48	49	63	62	55	51
Ethnicity	Caucasian	Caucasian	Caucasian	Caucasian	Caucasian	Caucasian
Clinical neurological manifestation	Balint’s syndrome	Neglect, apraxia, hemianopsia	Ataxia, dysarthria	Ataxia, gait instability, dysarthria	Ataxia, dysarthria	Apraxia, aphasia
Location of MRI lesions	Parietooccipital and inner capsule	Parietooccipital	Cerebellum, pons, medulla oblongata	Cerebellum, pons	Thalamus, capsula interna, precentral gyrus	Pons, crus cerebri, bifrontal, parietotemporal
Time from onset to diagnosis of PML (months)	15	3	7	1	3	1
JCV PCR in CSF (copies ml^−1^)	20	260	Not performed	Positive	129	720
JCV PCR detection threshold (copies ml^−1^)	10	10	N.a.	N.a.	100	100
PML diagnosis	Definite	Definite	Definite	Definite	Definite	Definite
Sarcoidosis diagnosis	Bioptically confirmed (lymph node), Stage II	Probable, Stage II	Probable, Stage II	Bioptically confirmed (lung), Stage I	Bioptically confirmed (lung), Stage II	Bioptically confirmed (lung), Stage II
Diagnosis of sarcoidosis	Prior	Concomitant	Retrospectively	Prior	Concomitant	Prior
Immunosuppressive medication when PML was diagnosed	None	None	None	50 mg b.i.d. AZA	None	20 mg q.wk. MTX, 10 mg q.d. PRED
CD4^+^ T-cell count at diagnosis (cells µl^−1^)	140	165	205	174	231	77
Course of PML after baseline	Progressive	Progressive	Stable	Stable	Progressive	Progressive
Treatment with infliximab	Yes	Yes	No	No	Yes	Yes
Last clinical FU after PML diagnosis (months)	84	78	103	44	46	–
CD4^+^ T-cell count at last FU (cells µl^−1^)	221	395	127	99	272	–
Clinical course of PML at last follow-up	Improved	Improved	Stable	Stable	Improved	Died

AZA, azathioprine; FU, follow-up; LN, lymph nodes; MTX, methotrexate; PRED, prednisolone; Stage I, lymphadenopathy; Stage II, lymphadenopathy and parenchymal disease; Stage III, parenchymal disease; Stage IV, pulmonary fibrosis.

^a^ Retrospectively identified.

^b^ At time point of first symptoms.

All patients with PML and concomitant sarcoidosis were male and the median age of onset was 53 years (range between 48 and 63 years). Presentation of PML symptoms correlated to the occurrence of lesions in the central nervous system and ranged from cerebellar symptoms to hemiparesis and Balint’s syndrome. Uniformly, PML lesions showed reduced signal intensity in T_1_-weighted scans and increased signal intensities in T_2_-weighted scans. Only Patient #2 showed gadolinium enhancement in T_1_-weighted scans as a rim-like structure. While standard diagnostics did not reveal any diagnosis in all of the patients, brain biopsy was performed and led to the diagnosis of PML which could be subsequently confirmed by JCV DNA in CSF of all cases in whom PCR was done (five patients). The median time from PML symptom onset to diagnosis was 3 months (range 1–15 months).

Three patients (Patients #1, #4, #6) already had a prior diagnosis of systemic sarcoidosis before the development of PML with a median time of 11 years (range 4–11 years) between the manifestation of sarcoidosis and PML. In two patients (Patients #2 and #5), sarcoidosis was identified during work-up for underlying immunodeficiency after diagnosis of PML. In one patient, sarcoidosis was diagnosed retrospectively after PML diagnosis (Patient #3). He was identified retrospectively by analysis of patients with bioptically confirmed PML and had probable systemic sarcoidosis upon re-evaluation but refused lymph node biopsy. In four patients (Patients #1, #4, #5 and #6), sarcoidosis was confirmed by histology of mediastinal lymph node or lung biopsy showing non-caseating granulomas (Fig. 1C). One patient refused biopsy (Patient #2), but radiological and clinical signs were strongly reminiscent of sarcoidosis.^4^ In two patients (Patients #1 and #2), FDG-PET scan was performed and showed increased tracer uptake of lymph nodes. Two patients were under immunosuppressive therapy (Patient #4: 50 mg b.i.d. azathioprine; Patient #6: 20 mg q.wk. methotrexate and 10 mg q.d. prednisolone) at the time of developing first PML symptoms, one patient (Patient #1) had not taken immunosuppressive therapy for 10.5 years and three patients (Patients #2, #3, #5) never received immunosuppressive therapy before developing PML symptoms. Serum sIL2R was elevated in four of the six patients (Patients #1, #2, #3 and #5) (Fig. 1D). Angiotensin-converting-enzyme (ACE) was measured in five patients but only slightly elevated in one of them (Patient #2; 75.2 I.E. l^−1^, reference <70.0 I.E. l^−1^). In all PML patients, other reasons for immunodeficiency, e.g. malignancies, asplenia, primary immunodeficiency, such as hypogammaglobulinaemia or HIV could be excluded and none of them had any other opportunistic infection in the past.

### Immune cell characterization

**Figure 2:**
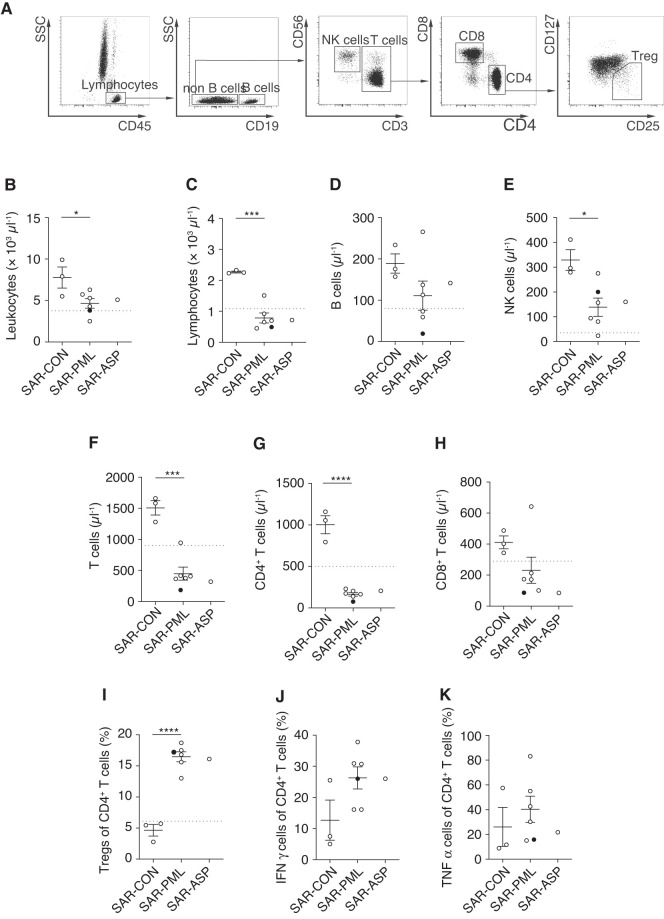
**Sarcoidosis patients with opportunistic infections show changes of T cell subset frequencies.** (**A**) Gating strategy for FACS analysis. Absolute numbers of (**B**) leukocytes [*T* (7) = 2.64, *P* = 0.033], (**C**) lymphocytes [*T* (7) = 6.30, *P* = 0.0004], (**D**) B cells [*T* (7) = 1.45, *P* = 0.191], (**E**) NK cells [*T* (7) = 3.14, *P* = 0.016], (**F**) T cells [*T* (7) = 6.18, *P* = 0.0005], (**G**) CD4^+^ T cells [*T* (7) = 10.74, *P* < 0.0001], (**H**) CD8^+^ T cells [*T* (7) = 1.42, *P* = 0.199], frequency of (**I**) regulatory T cells (Tregs) [*T* (7) = 8.89, *P* < 0.0001], (**J**) IFN-γ^+^ CD4^+^ T cells [*T* (7) = 2.02, *P* = 0.0827] and (**K**) TNF-α^+^ CD4^+^ T cells [*T* (7) = 0.77, *P* = 0.4685]. Grid lines show the lower reference limit for healthy controls (leukocytes >3800 µl^−1^, lymphocytes >1100 µl^−1^, B cells >80 µl^−1^, NK cells >35 µl^−1^, T cells >900 µl^−1^, CD4+ T cells >500 µl^−1^, CD8+ T cells >280 µl^−1^) or mean of 10 healthy controls for Treg frequency (6.1% ± SEM). SAR-CON (*n* = 3): control sarcoidosis patients without any opportunistic disease; SAR-PML (*n* = 6): sarcoidosis patients with PML; SAR-ASP (*n* = 1): sarcoidosis patient with aspergilloma; data from SAR-PML Patient #6 who received corticosteroids at blood sampling is presented as a filled circle. Data are presented as mean values ± SEM. Statistical analysis was performed by unpaired two-tailed Student’s *t*-test. *P < 0.05, **P < 0.01, ***P < 0.001, ****P < 0.00.

The finding of sarcoidosis-induced changes in T cell subset frequencies in Patient #1 prompted us to ask whether this is a unifying phenotype in sarcoidosis patients with PML. We used flow cytometry (for the gating strategy see Fig. 2A) to analyse the immune cell compartment in the blood of patients with PML and concomitant systemic sarcoidosis (SAR-PML) and compared it to patients with sarcoidosis but without PML or other opportunistic infections (SAR-CON). One SAR-PML patient (Patient #6) was initially treated for suspected neurosarcoidosis and was still under tapering corticosteroids at the time point of blood sample collection (7.5 mg q.d. prednisolone), whereas five patients did not receive any immunosuppressive medication at the time of analysis. Control patients (SAR-CON, *n* = 4) were diagnosed with definite systemic sarcoidosis (bioptically confirmed), age-matched (SAR-PML mean 55.67 ± 6.53 SD years; SAR-CON mean 47.75 ± 19.69 SD years; unpaired two-tailed Student's *t*-test [*T* (8) = 0.81, *P* = 0.44]) yet more often female (75% versus 0%; *χ*^2^ test, *P* = 0.011). One of the SAR-CON patients (Patient #3) had active sarcoidosis with increased tracer uptake of mediastinal, supraclavicular and paraaortal lymph nodes in FDG-PET scan with additional neurosarcoidosis, the other two had inactive sarcoidosis. We additionally included one patient with active sarcoidosis who was diagnosed with retroorbital aspergilloma (SAR-ASP) 7 years before inclusion in our study, which stabilized after 1 year of specific aspergilloma treatment. Neither the controls (SAR-CON) nor the patient with aspergilloma (SAR-ASP) was under any immunosuppressive medication at the time point of blood sampling. Characterization of control patients with sarcoidosis (SAR-CON) is shown in Table 2. For the reference of Treg frequencies, we also included 10 healthy volunteers (seven males and three females) without neurologic or inflammatory conditions.

**Table 2:** Sarcoidosis control cohort characteristics

Sex	Patient 1	Patient 2	Patient 3	Patient 4
	Male	Female	Female	Female
Age (years)^a^	49	36	31	75
Ethnicity	Caucasian	Caucasian	Caucasian	Caucasian
Sarcoidosis diagnosis	Stage II	Stage II	Neurosarcoidosis	Stage III/IV
Opportunistic disease	None	None	None	Aspergilloma

Stage I, lymphadenopathy; Stage II, lymphadenopathy and parenchymal disease; Stage III, parenchymal disease; Stage IV, pulmonary fibrosis.

^a^ At time point of inclusion.

We detected a significant reduction in overall numbers of leukocytes (*P* = 0.033, Fig. 2B) and lymphocytes (*P* = 0.0004, Fig. 2C) in the SAR-PML patients in comparison to the SAR-CON patients. No differences were detected in the number of B cells (*P* = 0.191, Fig. 2D) but in the numbers of NK cells (*P* = 0.016, Fig. 2E) and T cells (*P* = 0.0005, Fig. 2F) in the six SAR-PML patients in comparison to the SAR-CON patients. Specifically, the number of CD4^+^ T cells (*P* < 0.0001, Fig. 2G) was most significantly reduced. In contrast, the numbers of CD8^+^ T cells (*P* = 0.199, Fig. 2H) were not changed. Although there was no difference in Tregs numbers (*P* = 0.088, Supplementary Fig. 1), Treg frequencies (*P* < 0.0001, Fig. 2I) were significantly increased in SAR-PML patients. There was also a trend to higher frequencies of IFN-γ^+^ CD4^+^ T cells (*P* = 0.0827, Fig. 2J) and TNF-α^+^ CD4^+^ T cells (*P* = 0.4685, Fig. 2K) in comparison to SAR-CON. Comparisons to reference values were not possible as the cytokines were not measured in the healthy controls. Of note, changes in immune cell frequencies were also detectable in the patient with a history of opportunistic aspergilloma (SAR-ASP), which was indistinguishable from the SAR-PML group. These changes were independent of receiving immunosuppressive medication at the time point of analysis.

### Treatment and follow-up

In four of the prospectively followed patients (Patients #1, #2, #5, #6), PML rapidly progressed during 4 weeks of follow-up, which became apparent by severe clinical worsening and an increase in lesion volume in T_2_-weighted scans. As we detected a profound change in T cell subset frequencies in SAR-PML patients that could explain their predisposition to PML, we reasoned that treating the underlying sarcoidosis with specific immunotherapy instead of systemic immune suppression might restore immune cell frequencies and improve PML. Usually, active sarcoidosis is initially treated with corticosteroids followed by corticoid-sparing therapy, e.g methotrexate or azathioprine, both blocking proliferation of T cells,^14^ which would further suppress PML-directed immune responses. In contrast to broadly acting immunosuppressants, another frequently used therapy for sarcoidosis is infliximab,^4^ a chimeric monoclonal antibody directed against TNF-α. Since IFN-γ and TNF-α production by CD4^+^ T cells is hardly inhibited by Tregs in active sarcoidosis patients,^12^ we considered infliximab treatment as a chance for reducing inflammation, counteracting changes of immune cell frequencies and thereby ameliorating PML.

**Figure 3:**
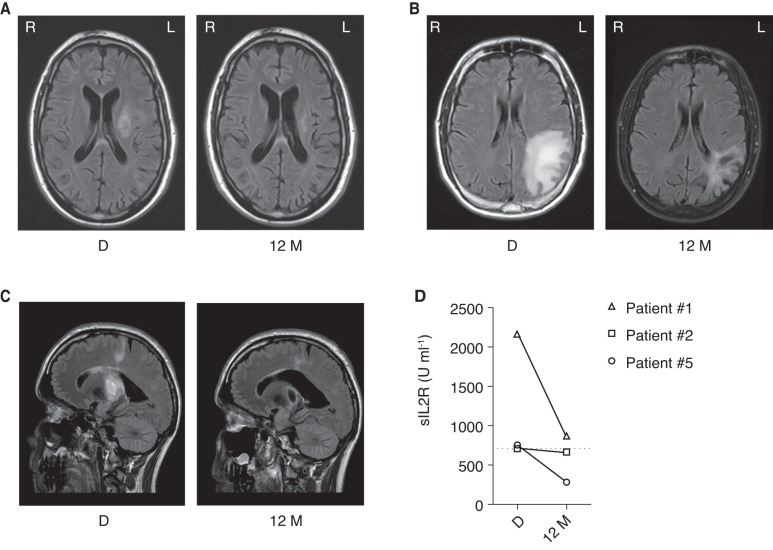
**Regression of MRI lesions and serum sIL2R with infliximab therapy.** MRI T_2_-weighted scans of (**A**) Patient #1, (**B**) Patient #2 and (**C**) Patient #3 at the time of diagnosis (**D**) and after 12 months of treatment with infliximab (12 M) is shown. (**D**) Serum sIL2 receptor (sIL2R) measurements after 12 months of treatment with infliximab (12 M) in comparison to time of diagnosis (**D**) in three patients with sarcoidosis and PML [*T* (2) = 1.65, *P* = 0.240, n.s.]. Grid line showing the upper limit of the reference range at <710 U ml^−1^. Statistical analysis was performed by paired two-tailed Student’s *t*-test.

An informed decision with the four patients for an individualized treatment with the goal to improve PML was reached. We initiated a treatment with infliximab (5 mg per kg body weight, every 6–8 weeks) in all four patients where PML rapidly progressed and followed them every 8 weeks. Three patients clinically stabilized at the first follow-up and slightly improved continuously during treatment. JCV became undetectable in the CSF after 1 year of treatment (detection limit 10 copies ml^−1^ for Patients #1 and #2 and 100 copies ml^−1^ for Patient #5). Similarly, MRI of these three patients stabilized after the first infusion and showed a continuous reduction in lesion size during treatment with an apparent reduction of lesions after 1 year of treatment with infliximab (Fig. 3A–C). We could detect faint gadolinium enhancement in the MRI of Patient #5 8 weeks after treatment initiation; however, as the patient continuously improved clinically, we did not suspect an overt immune reconstitution inflammatory syndrome. The enhancement disappeared 8 weeks later. The gadolinium enhancement in the MRI of Patient #2 at diagnosis was not detectable anymore during treatment. Improvement was paralleled by serum sIL2R normalization in two patients (Patients #2 and #5) and a marked decrease in the other patient (Patient #1) (Fig. 3D, *P* = 0.240), measured 1 year after therapy started. FDG-PET scan was performed in two patients (Patients #1 and #2) after 6 months of treatment and did not show anymore tracer uptake in lymph nodes.

**Figure 4:**
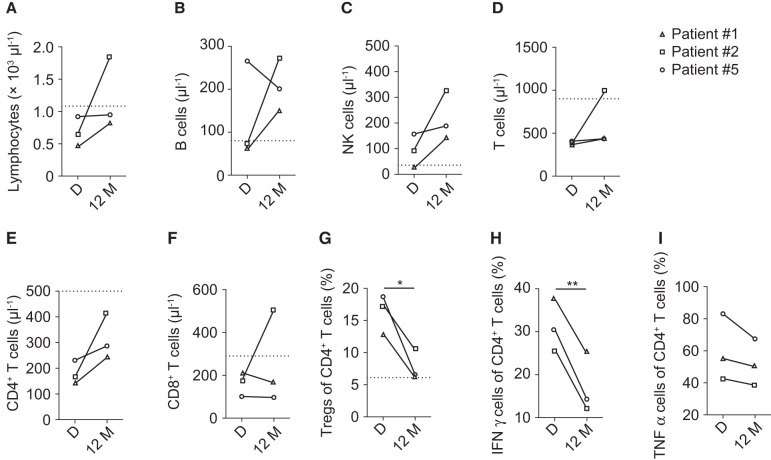
**T cell subset frequencies in sarcoidosis patients with PML change under infliximab treatment.** Absolute numbers of (**A**) lymphocytes [*T* (2) = 1.53, *P* = 0.265], (**B**) B cells [*T* (2) = 0.98, *P* = 0.428], (**C**) NK cells [*T* (2) = 2.17, *P* = 0.162], (**D**) T cells [*T* (2) = 1.27, *P* = 0.332], (**E**) CD4^+^ T cells [*T* (2) = 2.33, *P* = 0.145], (**F**) CD8^+^ T cells [*T* (2) = 0.77, *P* = 0.518] and frequency of (**G**) Tregs [*T* (2) = 5.10, *P* = 0.036], (**H**) IFN-γ^+^ CD4^+^ T cells [*T* (2) = 13.84, *P* = 0.005] and (**I**) TNF-α^+^ CD4^+^ T cells [*T* (2) = 2.20, *P* = 0.159] are shown. Grid lines show the lower reference limit for healthy controls: lymphocytes >1100 µl^−1^, B cells >80 µl^−1^, T cells >900 µl^−1^, NK cells >35 µl^−1^, CD4^+^ T cells >500 µl^−1^, CD8^+^ T cells >280 µl^−1^ or mean of 10 healthy controls for Treg frequency (6.10% ± SEM). *D* = time of diagnosis (*n* = 3), 12 M = 12 months (*n* = 3) of treatment. Data are presented as paired individual values. Statistical analysis was performed by paired two-tailed Student’s *t*-test. *P < 0.05, **P < 0.01.

We compared the immune profile before the start and after 12 months of infliximab therapy. Total numbers in lymphocytes (*P* = 0.265, Fig. 4A), B cells (*P* = 0.427, Fig. 4B), NK cells (*P* = 0.162, Fig. 4C), T cells (*P* = 0.332, Fig. 4D), CD4^+^ T cells (*P* = 0.145, Fig. 4E) and CD8^+^ T cells (*P* = 0.518, Fig. 4F) tend to increase, whereas Treg frequency significantly decreased (*P* = 0.036, Fig. 4G). Furthermore, percentages of IFN-γ-producing CD4^+^ T cells significantly decreased (*P* = 0.005, Fig. 4H) with treatment and TNF-α-producing CD4^+^ T cells also decreased, although not significantly (*P* = 0.159, Fig. 4I). Two patients (Patients #1 and #5) are still under infliximab treatment but with prolonged time intervals of 10 and 30 weeks between the infusions. In one patient (Patient #2), infliximab treatment was discontinued after 12 months due to rapid clinical and radiological improvement and the absence of detectable JCV in CSF. Until the last follow-up (median time from PML diagnosis until last clinical follow-up 78 months, range 46–84 months), all three patients remained radiologically stable without signs of PML activation and still slightly improved clinically.

Patient #6 was initially misdiagnosed with neurosarcoidosis and had received methotrexate and corticosteroids before the brain biopsy confirmed PML. At the time of diagnosis and start of infliximab treatment, he already showed severe neurological symptoms and the lowest CD4^+^ T cell count (76.9 µl^−1^). He died 9 weeks after treatment initiation due to aspiration pneumonia.

In one patient (prospective Patient #4), PML spontaneously stabilized clinically and radiologically without any signs of reactivation (time from PML diagnosis until last clinical follow-up 44 months). In Patient #3 (retrospectively), a vaccination with JCV capsid protein VP1 and experimental treatment with IL-7 was performed^15^ and PML stabilized (time from PML diagnosis until last clinical follow-up 103 months). The experimental approach was based on the idea that interleukin-7 (IL-7) is crucial for homeostatic T cell proliferation and restoration of T cell function, which can then allow an appropriate immune response against the VP1 protein in immunocompromised patients.

The experimental treatment was done before we retrospectively diagnosed him with sarcoidosis. However, in comparison to the infliximab-treated patients, the CD4^+^ T cell count at last follow-up (median time from PML diagnosis until last the follow-up and CD4^+^ T cell determination 70 months, range 40–92 months) was lower in these two patients in comparison to the infliximab-treated patients (see Table 1).

In summary, our data show an association of stopping progressive PML in three of four patients with infliximab treatment of their concomitant sarcoidosis. Improvement was paralleled by a decrease in systemic Treg expansion. One patient died shortly after treatment initiation most likely due to preexisting severe PML and not due to side-effects of medication. Together, infliximab seems to be a promising therapy to treat sarcoidosis that improves normal immune functions without affecting JCV-specific immune response.

## Discussion

Here, we show that active sarcoidosis can cause changes in immune cell frequencies, which can lead to PML predisposition and other opportunistic infections. Targeted treatment of underlying sarcoidosis with TNF-α inhibitors has the potential to repress sarcoidosis burden and thereby might partially normalize immune cell frequencies and halt PML progression in these patients.

Opportunistic infections, e.g. mycobacterial infections, cryptococcosis or aspergillosis, have been reported to occur in sarcoidosis patients^16–18^ as well as the simultaneous occurrence of sarcoidosis and PML. Often this was accompanied by immunosuppressive therapy, but several case reports of PML in untreated sarcoidosis patients can be found.^5,7,8^ It was also shown that the CD4^+^ T cell count was reduced in these patients.^5,8^ In our cohort, four from six PML patients with sarcoidosis were not under immunosuppressive therapy at the time of developing first PML symptoms. All patients showed a significant reduction of CD4^+^ T cell count and increased frequencies of Tregs, whereas there was no difference in the numbers of Tregs. However, Treg frequencies in relation to conventional T cells better reflect the overall suppressive capacity within the CD4 T cell compartment.^12^ Our results are in line with reports showing these changes predominantly in active sarcoidosis patients without PML, whereas this was not observed in any inactive sarcoidosis patients.^12^ The time from diagnosis of sarcoidosis to diagnosis of PML varies within our cohort. Sarcoidosis has active phases in which granulomas form and grow and inactive phases.^4^ Based on the results of our diagnostical work-up, we assume that all SAR-PML were in an active phase of sarcoidosis at the time of developing PML symptoms.

It was further shown that the expanded Tregs in active sarcoidosis patients did not completely inhibit TNF-α and IFN-γ production of CD4^+^ T cells and thereby were not able to control granuloma formation.^12^ Although the SAR-PML patients had higher frequencies of Tregs than the SAR-CON, there was a trend for higher frequencies of IFN-γ^+^ CD4^+^ T cells and TNF-α^+^ CD4^+^ T cells, which could indeed indicate an impaired suppressive capacity of Tregs on IFN-γ and TNF-α secretion resulting in sustained granuloma formation. However, the Tregs exhibited an antiproliferative effect on CD4^+^ T cells, which were overly active in sarcoidosis patients. Thus, active sarcoidosis can put patients at risk for T cell subset frequency changes and consequently the developing of PML or other opportunistic infections, which needs to be taken into consideration when caring for these patients.

All PML patients in our cohort were male and roughly the same age, whereas the patient with an aspergilloma was female. It is still a matter of debate whether there is a higher prevalence of sarcoidosis in males or females.^4,19^ Another case series of PML in sarcoidosis patients also reported more males but there was an equal sex ratio when analysing 20 additional cases.^5^

The reported fatality rate of PML in sarcoidosis patients with a mean observation time of 17.85 months is 57%.^5^ In our cohort, we have a low fatality rate of 16%. One patient (Patient #4) showed a stabilized PML disease course without treatment, although continuous low CD4^+^ T cell numbers. Spontaneous stabilization or recovery of PML in sarcoidosis patients has been reported.^5,^^20^ These stabilizations could be the result of inactivity of sarcoidosis or a containment of JCV by neutralizing antibodies or CD8^+^ T cells. In comparison to PML, cases in the context of severe immunodeficiencies such as HIV, the JCV copy numbers in the CSF were low in our cohort. As PML prognosis is associated with JCV viral load in the CSF,^21,22^ this could explain, why the outcome in our cohort is better than previously reported.

Four patients of our cohort showed rapidly progressing PML symptoms with increasing lesion sizes in MRI scans. Although there are a few cases using off licence drugs in PML,^3,15,23^ until now there is no specific approved PML medication. Usually, the most promising approach in PML treatment is removing the cause of immune deficiency. Therefore, we chose to treat the sarcoidosis and hypothesized to thereby be able to restore the immune response and halt PML. In order to avoid a potential aggravation of CD4^+^ T cell reduction, we refrained from using corticosteroids or a purine or pyrimidine inhibitor.^14^ By using infliximab, we were able to bypass this problem. Moreover, TNF-α plays a key role in granuloma formation^24^ as it mediates an excessive local T cell activity and Treg expansion with subsequent antiproliferative activity on CD4^+^ T cells.^12,25^ TNF-α downregulates the suppressive capacity of Tregs but also contributes to their accumulation.^26^ It was also shown that Tregs highly express TNF receptor 2 (TNFR2), which is further increased in sarcoidosis patients.^27^ By using infliximab, we anticipated that by inhibiting or preventing the binding of TNF-α to its receptor, we could reduce the activity of granuloma formation and the corresponding immune cell frequency changes.^28^ This is supported by a case series of sarcoidosis patients with CD4^+^ T cell lymphopaenia that showed a significant increase in absolute lymphocyte and CD4^+^ T cell counts after several weeks of treatment with infliximab.^29^ Indeed, we could observe a trend of increased CD4^+^ T cells and a significant reduction of Treg frequencies and IFN-γ-producing CD4^+^ T cells under the treatment with infliximab, which could indicate an improved suppressive capacity of Tregs on IFN-γ secretion. As we have not measured the frequencies in healthy controls and reference values differ between laboratories, it is unclear whether the reduction represents normalization, although all the frequencies 12 months after starting with infliximab treatment are within a reported reference interval.^30^

Baseline soluble TNFR2 in serum and TNFR2 expression on Tregs was higher in sarcoidosis patients of infliximab responders than in non-responders.^27^ Hence, it could be useful to analyse these markers in sarcoidosis patients that develop PML and compare the treatment responses. Whether TNFR2 expression is connected with the treatment failure in the patient who died after 9 weeks of therapy (Patient #6) is unclear. However, this patient already presented with a high lesion burden at the time of diagnosis and showed higher JCV copy numbers in the CSF in comparison to the other patients. He was initially misdiagnosed with neurosarcoidosis and received methotrexate and corticosteroids which might have further worsened JCV control.

To the best of our knowledge, this is the first report of an infliximab treatment of PML in sarcoidosis patients. PML cases under TNF-α antagonist treatment are rare and usually accompanied by other immunosuppressive drugs.^31,32^ We are not aware of any case in a sarcoidosis patient in which PML developed under TNF-α antagonist treatment. Furthermore, T cell response to JCV is independent of TNF-α^33^ and TNF-α stimulation might rather contribute to JCV transcription.^34^

Indeed, infliximab treatment was associated with a continuous improvement of symptoms and decreased MRI lesions in three treated PML patients. Notably, also serum sIL2R decreased in these patients. However, serum sIL2R was only elevated in four of six SAR-PML patients, which agrees with the fact that serum sIL2R is not necessarily elevated in active sarcoidosis patients,^35–37^ although still more sensitive and specific than ACE.^38^ IL2R is expressed at the surface of activated T cells and released into the serum. Therefore, sIL2R is a general marker for T cell activation. As sIL2R could be elevated in all CD4^+^ T cell-mediated inflammatory processes, it is not specific for diagnosing sarcoidosis, though it might still be useful in assessing the disease activity of sarcoidosis.^39^ IL2R binds IL2, which is a cytokine released by CD4^+^ T cells and induces proliferation of T cells. Additionally, IL2R signalling is crucial for the maintenance of Treg survival^40,41^; however, whether the decreased frequencies of Tregs after infliximab treatment are also mediated by decreased IL2 needs to be investigated.

Our study has some limitations. Besides small samples sizes in both groups, a long-term follow-up of the SAR-CON patients was not available. Though, the Treg frequencies of our SAR-CON patients were within the same range as Treg frequencies in a previously reported sarcoidosis cohort.^12^ Due to the progression of four patients, we decided to treat all of them with infliximab. Consequently, we were lacking a control group of progressive SAR-PML patients in which the spontaneous disease course could have been monitored.

Still, already this small cohort study indicates that a systemic change of frequencies of T cell subsets in sarcoidosis constitutes a risk factor for the development of PML or other opportunistic diseases and should be actively investigated in sporadic PML patients. Thus, analysis of CD4^+^ T cells and Tregs should be performed in sarcoidosis patients with overt opportunistic infections. Moreover, as PML and lymphopaenia could also occur in other autoimmune rheumatological diseases (e.g. systemic lupus erythematosus)^42,43^ without prior immunosuppressive treatment, PML should be considered as a differential diagnosis in patients with neurological symptoms. Finally, targeted treatment of underlying sarcoidosis to restore lymphocytopaenia might be a valuable option to improve PML and other opportunistic infections in the future. However, as the sample size in this study is small and the utility of infliximab is not proven, a multi-centre, open-label, register-based treatment trial of infliximab in PML patients with sarcoidosis could help to further support our observations.

## Supplementary Material

fcab292_Supplementary_Data

## Abbreviations

ACE =: angiotensin-converting-enzyme
FDG-PET =: fluorodeoxyglucose-PET
IFN-γ =: interferon-γ
IL-2 =: interleukin-2
IL-7 =: interleukin-7
IRIS =: immune reconstitution inflammatory syndrome
JCV =: JC virus
PBMCs =: peripheral blood mononuclear cells
PML =: progressive multifocal leukoencephalopathy
SAR-ASP =: sarcoidosis patient with retroorbital aspergilloma
SAR-PML =: sarcoidosis patients with PML
SAR-CON =: sarcoidosis patients without PML
sIL2R =: soluble interleukin-2 receptor
TNF-α =: tumour necrosis factor-α
sTNFR2 =: soluble Tnfr2
TNFR2 =: Tnf receptor 2
Treg =: regulatory T cell
