# Supplementary material for: Treating sarcoidosis-associated progressive multifocal leukoencephalopathy with infliximab
Source: Brain Commun. 2021 Dec 16;4(1):fcab292. doi: 10.1093/braincomms/fcab292 (PMC8727989; doi:10.1093/braincomms/fcab292)
Supplement: fcab292_Supplementary_Data [file fcab292_supplementary_data.pdf]

Supplementary material

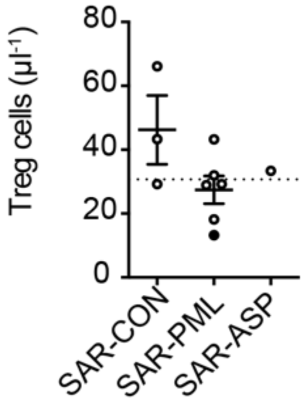

Supplementary Figure 1. Absolute numbers of Tregs in sarcoidosis patients. Absolute numbers of Tregs. Grid lines show the mean of Treg numbers from ten healthy controls. SAR-CON (*n* = 3): control sarcoidosis patients without any opportunistic disease; SAR-PML (*n* = 6): sarcoidosis patients with PML; SAR-ASP (*n* = 1): sarcoidosis patient with aspergilloma; data from SAR-PML patient #6 who received corticosteroids at blood sampling is presented as a filled circle. Data are presented as mean values ± s.e.m. Statistical analysis was performed by unpaired two-tailed Student's *t*-test.
